# Synthesis, Characterization and Decomposition of Potassium Jarosite for Adsorptive As(V) Removal in Contaminated Water: Preliminary Study

**DOI:** 10.3390/ijerph192315912

**Published:** 2022-11-29

**Authors:** Eduardo Cerecedo-Sáenz, Elías Hernández-Lazcano, Maythe J. González-Bedolla, Juan Hernández-Ávila, Raúl Rosales-Ibáñez, María del P. Gutiérrez-Amador, Ariadna Sánchez-Castillo, Alberto Arenas-Flores, Eleazar Salinas-Rodríguez

**Affiliations:** 1 Academic Area of Earth Sciences and Materials, Institute of Basic Sciences and Engineering, Autonomous University of the State of Hidalgo, Highway Pachuca-Tulancingo km. 4.5, Mineral de la Reforma, Pachuca 42184, Mexico; 2 Tissue Engineering and Traslational Medicine Laboratory, Faculty of Higher Education, Iztacala, National Autonomous University of Mexico, Tenayuca-Chalmita S/N, Cuautepec, Barrio Bajo, Alcaldía Gustavo A. Madero, Ciudad de Mexico 07239, Mexico; 3 Apan High School, Autonomous University of the State of Hidalgo, Highway Apan-Calpulalpan km. 8, Apan 43920, Mexico

**Keywords:** wastewater, arsenic, arsenic elimination, water depuration, jarosites

## Abstract

Jarosite-type compounds precipitated in the zinc industry for iron control can also incorporate arsenic and can be used for wastewater treatment for As elimination. According with the last, this work is related to arsenic incorporation at room temperature in decomposed potassium jarosite. The work began with the synthesis of the compound at 75 °C for 9 h using Fe_2_(SO_4_)_3_ and K_2_SO_4_ at a pH of 1.1. Once jarosite was obtained, solids were subjected to an alkaline decomposition using NaOH at pH 10 for 30 min, and then As was added to the solution as HAsNaO_4_ and the pH modified by adding HNO_3_ until it reached a value of 1.1. The initial, intermediate, and final products were wholly characterized by scanning electron microscopy (SEM) in conjunction with energy dispersive spectrometry (EDS), X-ray diffraction (XRD), Fourier transform infrared spectroscopy (FTIR), Raman spectroscopy (RS), and X-ray photoelectron spectrometry (XPS). The obtained results show that As(V) can be adsorbed by ionic exchange in the amorphous FeOH structure of decomposed jarosite and when pH decreased to 1.1, the compound recrystallized, incorporating up to 6% As on average, which is indicative that this process can be used to reduce As in contaminated waters.

## 1. Introduction

Decontamination is, in general, defined as the removal of hazardous material, and is used to reduce the potential of air or water containing chemical, biological, radiological, and nuclear (CBRN) agents [1]. In the eventuality of emergency situations, people need to be provided with an adequate supply of drinking water, which requires all aspects of detection, cleaning, purification, and treatment, including desalination, disinfection, and decontamination of water [2].

The presence of As in water represents a harmful contaminant in drinking water, aquatic life, agricultural activities, and so on, considering that this metalloid must have a maximum limit in discharge of 10 mg/L. In many countries today, the limit mentioned above is exceeded. In superficial water, As can be present as As(V) while deeper it can be found as As(III) [3,4,5]. For this reason, it is important to control the As content in water to protect the environment and to guarantee the quality of all kinds of water [6]. 

The use of lime-iron salt is a process that uses arsenite and arsenate to form a stable precipitate with the calcium ion, which is also insoluble in water. The process promotes the Fe^3+^ ion property of being a good adsorbent and allowing As precipitation after Fe^3+^ hydrolysis, reaching up to 0.28 mg/L, and with the additional use of H_2_O_2_ for the oxidation of Fe^2+^ and As(III), it could achieve As contents of up to 0.014 mg/L, and both are below the permitted limit [7,8,9,10].

On the other hand, some researchers have achieved conversion of more than 99% of S into AsS^3−^ by controlling the As:S ratio and other variables such as pH, and so on. In the same way, under strong acidic conditions, 99.9% of As can be removed from contaminated water by hydrogen sulfide injection into the liquid residue, leaving as little as 0.5 mg/L of As after two treatments [11,12,13].

Similarly, there are other methods such as adsorption, which uses two types of adsorbents, the first being metals and their alloys such as manganese oxide [14], activated alumina, and iron compounds [15]. The second category involves activated carbon [16], red mud, orange peel [17,18], coconut shell [18], carbon [19], eggshell [20,21], java plum seed, water chestnut shell, corn cob, tea waste, and pomegranate peel, among others [21]. These biosorbents were used for arsenate (As(V)) and arsenite (As(III)) removal from contaminated water, achieving As recoveries from 65 to 74% at pH values ranging from 4.1 to 7, and increasing adsorption up to 97% after four consecutive cycles of treatment [21,22,23]. Using the other method, performing the adsorption of As(V) on an Fe(III)-loaded sponge, resulted in fast adsorption of arsenate by an Fe(III)-loaded sponge in less than ten minutes [24].

Likewise, arsenic can be incorporated in minerals such as natroalunite and scorodite. For the natroalunite with As, it was determined that the rate of precipitation of this compound increases as temperature and ${\mathrm{As}}_{4}^{-3}$ concentration increases, and was practically completed in less than 30 min at temperatures between 180 and 200 °C. Maximum substitution of As in the natroalunite structure was approximately of 7–15% molar. Similarly, it was determined that As stability varied under different conditions, with the maximum stability occurring at pH 5–8, resulting in solubility of 0.01–0.05 mg/L in 24 h. These low rates of As liberation make this compound an adequate mineral phase for the blanketing of wastes containing great ${\mathrm{SO}}_{4}^{-2}/{\mathrm{AsO}}_{4}^{-3}$ ratios [25,26,27]. For the incorporation of As during the synthesis of scorodite, Fijita et al. [28,29] found that this process involves oxidation of ferrous ions in the presence of high concentrations of As(V). Synthesis was executed at 95 °C over 7 h, with pH of 2.0, ferric sulfate, and Fe:As at 1.5 ratio. Thereby, the jarosite-type compounds belong to the alunite group, of which the general formula is DG_3_(TO_4_)_2_(OH, H_2_O)_6_, where D are cations with a coordination number greater than or equal to nine, being monovalent (Na, K, Ag, H_3_O, etc.) or divalent (Pb, Sr, Ba, Ca, etc.). The G site is usually trivalent (Al, Fe). TO_4_ is dominated by one or more ${\mathrm{SO}}_{4}^{-2},{\;\mathrm{AsO}}_{4}^{-3}$, and ${\mathrm{PO}}_{4}^{-3}$. Therefore, jarosites are very common in acid mine drainage, acid sulfate soils, bioleaching systems, and also are precipitated in hydrometallurgical circuits for Fe elimination, where jarosite wastes after their neutralization are discharged in tailing dumps [30].

According to the above description, jarosites have been investigated to be proposed for the immobilization of arsenic in their structures. However, in some cases, both arsenical jarosites and scorodite provide only temporary storage for As in the environment because of the limited pH and redox conditions under which they are stable [30,31,32,33]. Generally, the synthesis of arsenical jarosites has been carried out at high temperatures and with long reaction times. For example, the system K_2_O-Fe_2_O_3_-As_2_O_5_- H_2_SO_4_-H_2_O was synthesized at 95 °C and 1 bar pressure, while the arsenical ammonium jarosite was synthesized at 94 °C over 24 h of reaction, obtaining an incorporation of As of up to 3.19%. In the same way, such jarosite was decomposed in alkaline media, showing that As remains in the amorphous FeOH structure obtained once the decomposition process finished [34,35], similar to what was described for the alkaline decomposition of arsenical sodium jarosite [36]. On the other hand, other authors found that there are different levels of As substitution in the structure of minerals from the alunite family, determining substitutions of 3–8.5% in weight for beudantite; 3.6% in weight for alunite; 2.8% in weight for natroalunite; and 1.6% in weight for jarosite, depending on the AsO_4_/TO_4_ ratio. Moreover, As liberation was also evaluated from alunite and natroalunite at pH 5–8 as aerobic and abiotic arsenic. In addition, a similar behavior was detected for jarosites at pH 8, and this phenomenon can be attributed to the stability of jarosite structures or even to the formation of other structures that can readsorb the As [37,38].

Finally, this work relates to the incorporation of As by cationic exchange in the structure of a decomposed potassium jarosite (FeOH) previously synthesized using the method described in other works [39,40]. The novelty of the present study relates to the viability of the incorporation of about 4.19% As(V) in a decomposed jarosite at room temperature in alkaline media (pH 10), before decreasing the pH to 1.1 immediately, at which point the crystalline structure of potassium jarosite was again observed. In this way, the results could be used for the study of heavy metal adsorption using jarosites precipitated in the zinc industry, that are discharged in dumps.

## 2. Materials and Methods

### 2.1. Synthesis of Potassium Jarosite

For this work, the potassium jarosite synthesis was executed using the modified method utilized in previous works [39,40]. The synthesis method followed the chemical dissolution–precipitation with a slight modification of parameters such as temperature, concentration, pH, and reaction time.

**Figure 1:**
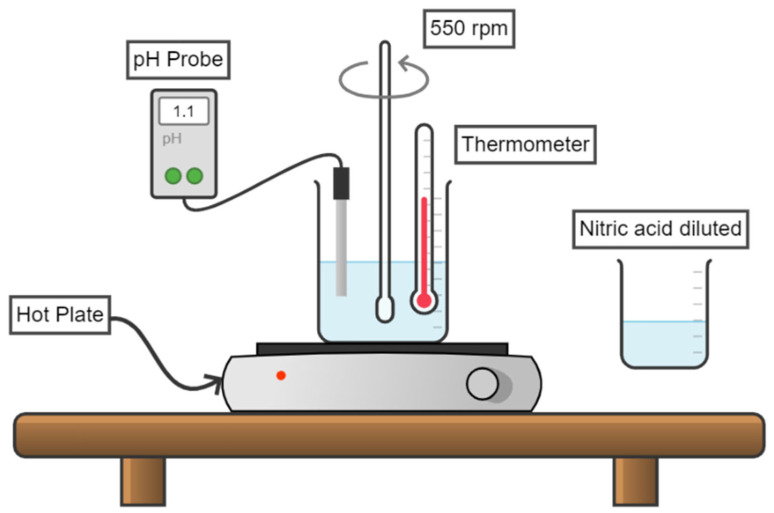
Experimental setup for synthesis of the potassium jarosite.

For the synthesis procedure, iron sulfate (Fe_2_(SO_4_)_3_) and potassium sulfate (K_2_SO_4_) were used as the source of the corresponding jarosite. In addition, sodium hydroxide (NaOH) and sulfuric acid (H_2_SO_4_) were used to adjust pH at the beginning of the synthesis stage. All chemicals were purchased from Sigma-Aldrich (St. Louis MO, USA) with a purity > 99%. Firstly, 0.15 M of Fe_2_(SO_4_)_3_ and 0.15 M K_2_SO_4_ were dissolved in a volume of 0.5 L of deionized water in a three-neck flask equipped with a pH measurement system, with the temperature maintained at 75 °C for 9 h of reaction at a pH value of 1.1, using a stirring rate of 550 s^−1^. Figure 1 shows the experimental setup for obtaining the jarosite powders.

After 9 h of reaction, brown precipitates were obtained, which were used for the subsequent stage of alkaline decomposition. Finally, precipitates were collected, filtered, washed (using deionized water at 60 °C), and dried in a muffle at 65 °C. The procedure was repeated until sufficient sample was produced for the next stages of decomposition and incorporation of As(V).

### 2.2. Alkaline Decomposition of Potassium Jarosite and Addition of As(V)

**Figure 2:**
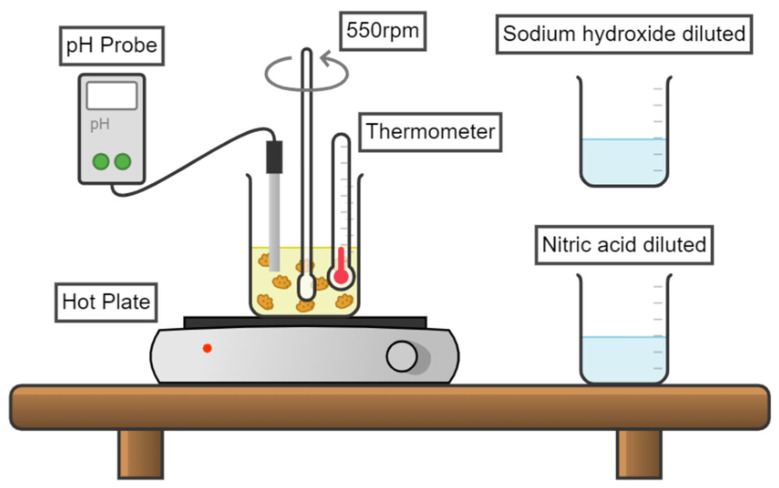
Experimental setup for the alkaline decomposition of potassium jarosite and incorporation of As(V).

Figure 2 shows the array for the decomposition of jarosite and the subsequent incorporation of arsenic.

For the alkaline decomposition of potassium jarosite, the experimental procedure was similar to that employed for decomposition of rubidium jarosite [41]. Firstly, 3 g of the potassium jarosite previously synthesized were placed in a volume of 100 mL of distilled water at 25 °C under mechanical stirring (550 s^−1^) in 1.5 M NaOH at a pH of 10.

The total decomposition reaction took place in 30 min, and at the end of this period of time, the As(V) was added as 0.03M HAsNaO_4_•7H_2_O (with purity of 98%) from Sigma-Aldrich (St. Louis, MO, USA). As(V) was added to the same solution where decomposition was performed, and immediately after the addition of As(V), drops of 2 M HNO_3_ were added until a pH value of 1.1 was reached. The intention of returning to the pH of jarosite formation was to carry out recrystallization of the decomposed compound and thus trap As(V) in that structure like ${\mathrm{AsO}}_{4}^{-3}$. All experiments were executed for 120 min, taking samples at determined time intervals (0, 10, 20, 30, 40, 50, 60, 70, 80, 90, 100, 110, and 120 min) to observe the evolution of As(V) incorporation during the process.

### 2.3. Material Characterization

All the synthetic jarosite powders, the jarosite already decomposed, and the final product containing the As(V) adsorbed into the jarosite structure were wholly characterized.

Foremost, X-ray diffraction (XRD) was employed to disclose the crystalline structure of the obtained compounds, and for this analysis, the powder technique was used with a Bruker D8 Discover diffractometer, using a CuKα = 1.5406 Å radiation source operating at 40 kV and 40 mA. All diffraction spectra were obtained in a 2θ range from 10° to 70° using an increment step size of 0.03°, to identify the phase compound and the crystalline structure of potassium jarosite. Finally, indexing of the obtained diffractograms was carried out with the software MATCH version 1.1 (developed by Crystal Impact, Bonn, Germany).

In addition, the morphological and punctual chemical analysis was performed with low-vacuum scanning electron microscopy (LV-SEM) in a JEOL JSM5900-LV machine equipped with an Oxford energy dispersive spectrometer (EDS) and operated at 20 kV.

Finally, to confirm the presence and relative amount of As(V) in the potassium jarosite powders, three different analytical techniques were used to characterize the samples of jarosite obtained after As(V) adsorption, and the aforementioned techniques are the following: (1) Total reflectance Fourier transform infrared (ATR-FTIR) was conducted using a Perkin-Elmer Frontier FTIR spectrometer brand RAINSHAW, model InVia, where 10 mg of sample was carefully placed on the crystal surface, and each obtained spectrum was recorded as absorbance under 75%. In addition, each spectrum was scanned between 4000 and 400 cm^−1^ wavelengths. (2) Raman spectroscopy using a HeNe laser (633 nm) at a power of 10%, with an exposure time of 60 s, and scanning from 100 to 3200 cm^−1^. (3) X-ray photoelectron spectroscopy (XPS) analysis was executed in a K ALPHA Surface Analysis machine (Thermo Scientific) which has a hemispherical (180°) analyzer of double approach and 128-channel detector with a base pressure of 2 × 10^−9^ mbar. The X-ray gun uses the Al monochromated Kα line (1486.6 eV) at 12 kV and 40 watts of power in an oval area with a diameter of 400 um, and it affects the sample with a relative angle of 30°. In erosion, a beam of argon ions accelerated at 3 kV with a power of 30 W is used, incident in an area of 1 × 2 mm concentric to the X-ray beam. The neutralizer generates a cloud of argon ions at close to 0 V of energy on the analyzed zone. XPS spectra were obtained under two conditions: in wide sweep (0–1350 V) with 1 eV/step, and the small windows mode with 0.1 eV/step, with step energy 50 eV.

## 3. Results

### 3.1. Synthesis of the Potassium Jarosite

**Figure 3:**
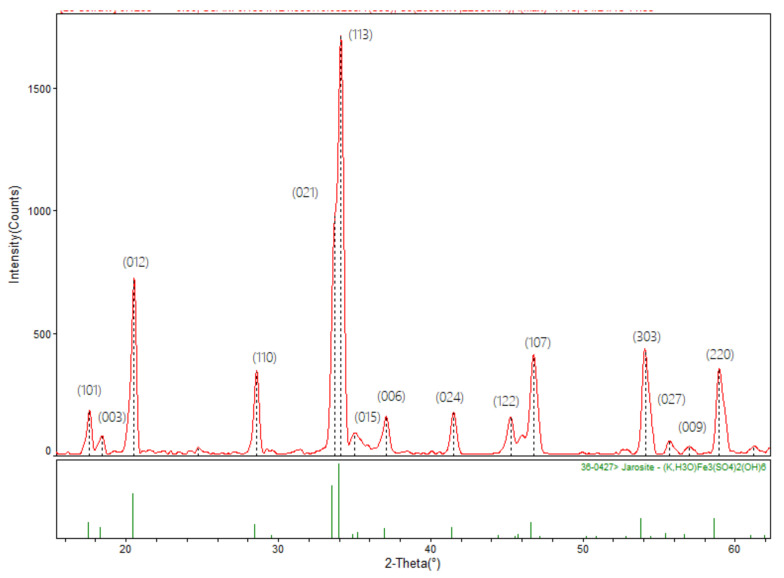
X-ray diffraction spectrum of the synthesized potassium jarosite (75 °C, 9 h, and pH 1.1).

For the first stage of this work, the obtained powders of potassium jarosite synthesized under the aforementioned conditions were characterized by XRD to disclose their principal mineralogical and crystalline characteristics. The X-ray diffraction pattern for the jarosite synthesized is shown in Figure 3. The pattern was indexed using the JADEv5 software (developed by Materials data JADE, Livermore, CA. USA)that corresponds to Powder Diffraction File (PDF) 36–0427, belonging to potassium-hydronium jarosite (K, H_3_O)Fe_3_(SO_4_)_3_(OH)_6_. These results confirm the formation of the corresponding jarosite exhibiting good crystalline structure.

On the other hand, the calculation of the average crystallite size of J-K-H was carried out using the Scherrer equation (Equation (1)), where k is a shape function having a value of 1, λ is the X-ray wavelength, FWHM is the average width of the experimental peak, and θ is the angle of incidence.

(1) $$\beta =\frac{k*\lambda }{\mathrm{FWHM}*\cos\theta }$$

Table 1 shows the data used for solving Equation (1), and so obtaining the crystallite size corresponding to J-K-H results in an average size of ~4.55 nm.

**Figure 4:**
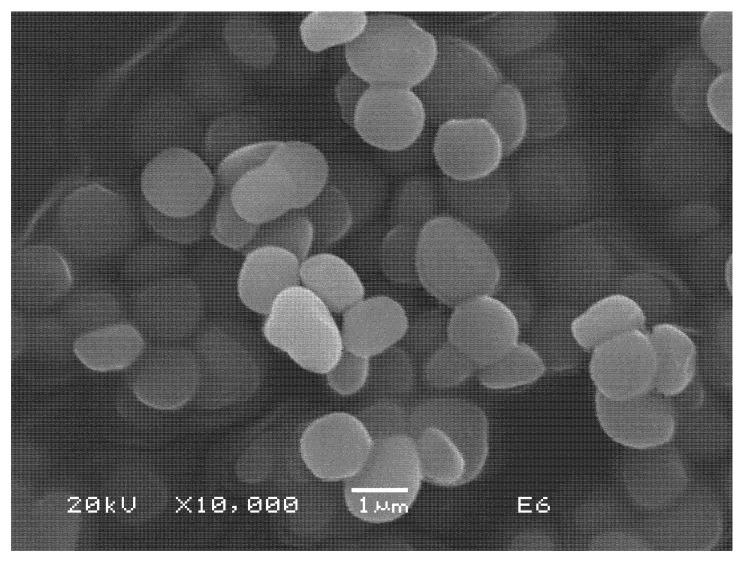
SEM (SE) images of potassium jarosite obtained in the synthesis stage (magnification of 10,000×).

In a similar way, characterization of synthesized jarosite by SEM-EDS showed small spherical particles of an average size of 1–2 μm, which could support the fact that As(V) can be incorporated into the decomposed structure and during the recrystallization stage, while Table 2 shows the average punctual composition of this jarosite compound obtained by energy dispersive spectrometry (EDS). These values, although obtained using a semiquantitative analysis, are representative due to all particles present in the sample being similar in morphology, and their particle size showing great homogeneity. In the same way, these results correspond to the synthetic potassium jarosite, where H_3_O + OH were obtained by difference. Figure 4 shows the distribution of particles obtained after synthesis of potassium jarosite; these were used for the decomposition and adsorption of As(V) into their crystalline structure due to the spherical morphology obtained and the particle sizes averaging 0.8 to 2 μm, parameters that make these kinds of particles adequate for As(V) adsorption, and their fast after-recrystallization stage.

### 3.2. Potassium Hydronium Jarosite Decomposition and Adsorption of As(V)

**Figure 5:**
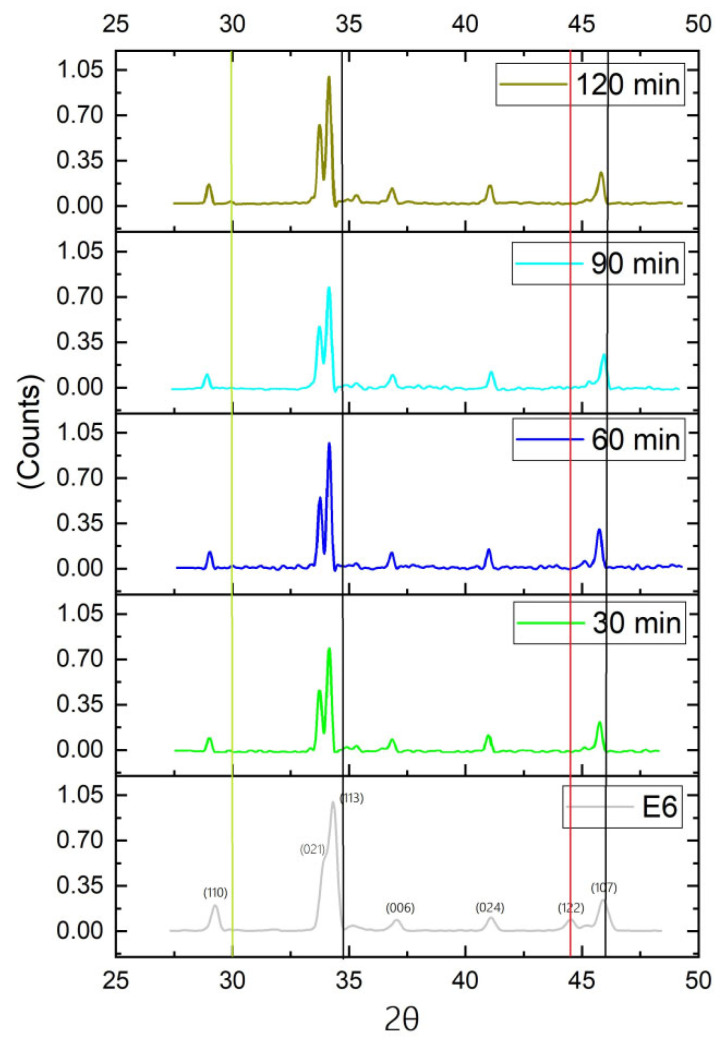
XRD spectra for potassium jarosite and the powder obtained after 30, 60, 90, and 120 min of reaction of incorporation of As(V).

After synthesis of potassium jarosite, it was decomposed in alkaline media, and As(V) was immediately added to the solution to promote its adsorption into the jarosite structure. The resulting powders were characterized to determine if As(V) was successfully incorporated. Figure 5 shows the XRD spectra obtained from the powder at different times during the As(V) adsorption process, in comparison to the original spectrum of jarosite without As(V).

Figure 5 exhibits a light displacement of the principal and secondary peaks to the left, which could be indicative of a change in the size of the structure due to the AsO_4_ incorporation in the jarosite structure. Further, the 30, 60, 90, and 120 min reactions exhibit a more marked plane (021), and all spectra corresponding to the incorporation of As(V) display the disappearance of the small peak (plane 122) that is to the left of the plane (017), which also confirms the change in structure due to the adsorption of AsO_4_. Black lines indicate that peaks are moving to the left as a consequence of As(V) adsorption, indicating the cell expansion at 44 (2θ degrees). The red line indicates that the peak of plane (122) tends to disappear in all jarosites where As was adsorbed, while the green line at 30 (2θ degrees) indicates an extra peak (double) promoted by the As(V) incorporation partially replacing the SO_4_.

**Figure 6:**
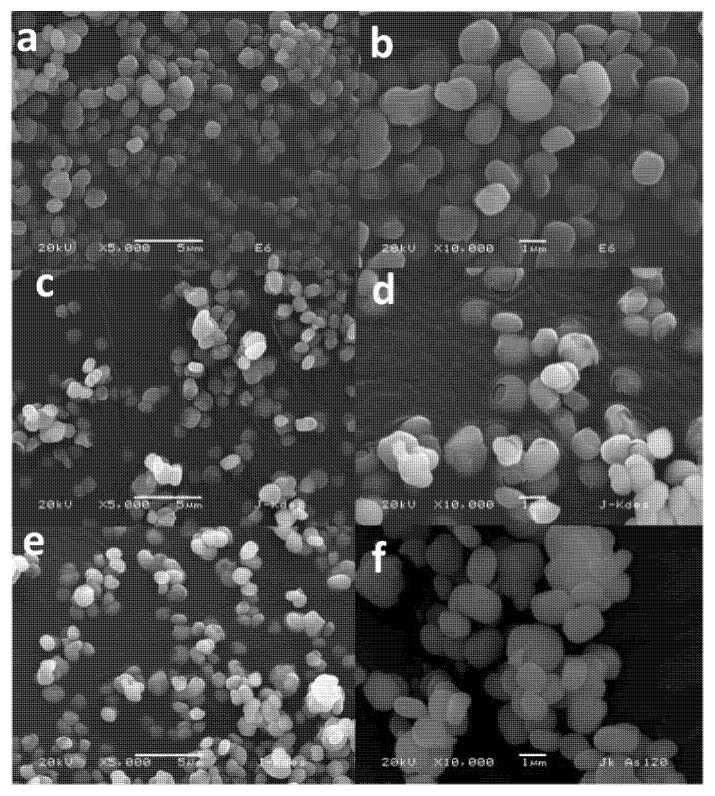
SEM (SE) images of: (**a**,**b**) synthesized potassium jarosite; (**c**,**d**) decomposed potassium jarosite; and (**e**,**f**) potassium jarosite with As(V) and recrystallized. Left and right column images are under magnifications of 5000× and 10,000×, respectively.

Further, particles of jarosite decomposed with NaOH, and those already recrystallized containing arsenic (V), were observed using SEM (SE) to disclose their morphological evolution. Figure 6 shows the images produced for the corresponding powders obtained. One can observe a solid structure for the initial potassium jarosite synthesized (Figure 6a,b) while the decomposed jarosite shows a broken structure (Figure 6c,d) where SO_4_ and K dissolved ionically into the solution, remaining solid in the amorphous and porous phase of FeOH. Finally, during incorporation of As(V) in the decomposed jarosite and its recrystallization, particles recovered their initial morphology (Figure 6e,f) as a compact structure.

Once recrystallization finished, the morphology of particles resembled that obtained after synthesis. Figure 6e,f show images of particles after 120 min of reaction of As(V) adsorption and display the recovery of particles (compared with Figure 6a,b), with respect to shape and size.

For the case of the EDS analysis, Table 3 shows the corresponding results obtained for synthesized jarosite and the powders obtained after 30, 90, and 120 min of As(V) adsorption reaction. It can be observed that after decomposition, As(V) adsorption occurred, increasing its content from 7.3, 8.48, and 8.47% for the corresponding reaction times prefixed, 30, 90, and 120 min, respectively (SEM-EDS semiqualitative and punctual analysis).

Furthermore, according to the obtained results from ATR-FTIR, more information was found regarding to adsorption of As(V) in the potassium jarosite structure. Likewise, it is known that in electromagnetic radiation, the IR region corresponds from 10 to 13,000 cm^−1^. However, only the mid-IR region (400–4000 cm^−1^) is used in conventional IR analysis, due principally to the fact that fundamental vibration or functional groups to be analyzed belong to this region [42].

**Figure 7:**
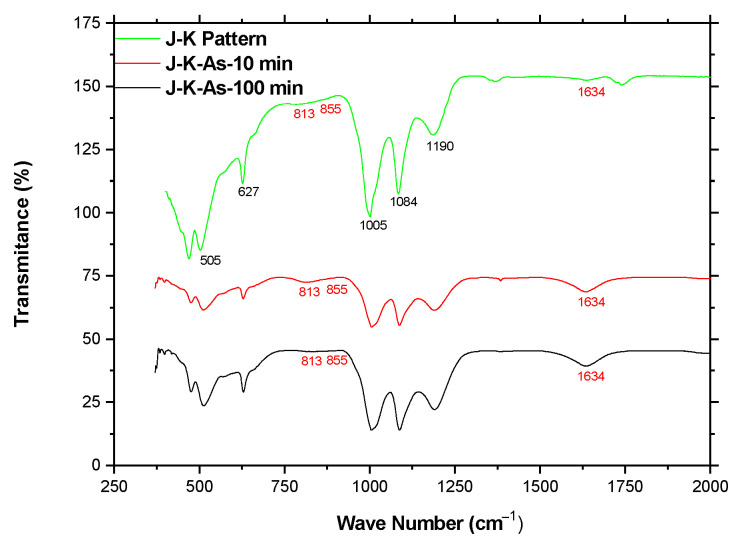
FTIR spectra of the potassium jarosite with As(V) compared to spectrum for J-K pattern.

Figure 7 shows the IR spectra for two samples of potassium jarosite with As(V) incorporation at 10 and 100 min of reaction compared with a pattern of the K-jarosite. It can be observed that both spectra (10 and 100 min) are quite similar in pattern, and both exhibit adsorption bands of comparable shape and position, principally where As(V) presence is described. According to the literature, peaks related to the presence of arsenate are between 813 and 855 cm^−1^, establishing that the peak at 828 cm^−1^ marks the presence of the functional group As-O [43], which corresponds to the *v*_1_ (As$O_{4}^{3-}$) and *v*_3_ (As$O_{4}^{3-}$) modes, respectively, and the peak that represents the substitution of arsenate for sulfate is at 1634 cm^−1^ [35]. For the present case, one can observe the minor presence of the mentioned groups, and also that in the case of substitution of arsenate for sulfate (1634 cm^−1^), this was more pronounced for the 100 min of As(V) reaction of adsorption. Additionally, peaks were identified that correspond to the absorption bands of *v*_3_ (double) and *v*_1_ vibrations for sulfates (1005, 1084, and 1190 cm^−1^) to the presence of ${\mathrm{SO}}_{4}^{2-}$ at 627 cm^−1^, and to the vibration of the octahedral coordination of FeO_6_ at 508 cm^−1^, and all were reported in previous work for ammonium-sodium jarosite with As(V) [35].

On the other hand, Raman spectroscopy analysis is a useful tool for finding the stretching and bending vibrations associated with the interactions of atoms in minerals and compounds and has been used as a complementary technique to disclose elements and compounds involved in synthetic and natural jarosites [32,33,34,35,36,37,38,39,40,41,42,43,44].

**Figure 8:**
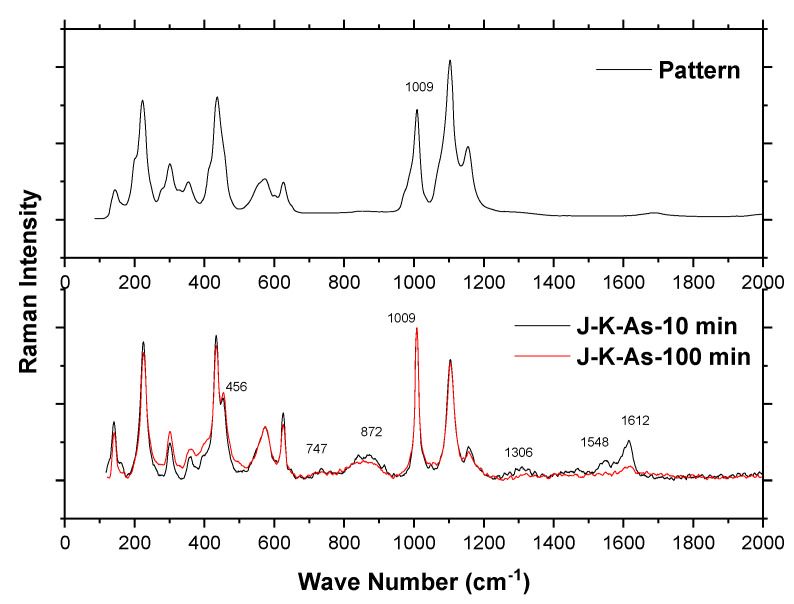
Raman spectra of the potassium jarosite with As(V) compared to potassium jarosite without As(V).

Raman spectra for the potassium jarosite with As(V) incorporated over 10 and 100 min are shown in Figure 8. There are peaks located at two regions, which correspond to the internal modes of the SO_4_ group (430–650 and 980–1200 cm^−1^) and Fe-O stretches (less than 440 cm^−1^), as was pointed out by other authors [44]. On the other hand, the literature has reported that the Raman spectra of the AsO_4_ stretching region is present in the range 750–950 cm^−1^ [45], as can be observed in Figure 8, in comparison with the pattern of jarosite without As(V). Further, theoretical values for free arsenate (with tetrahedral symmetry) can be found in 887 cm^−1^ (*v*_3_*)* and 463 cm^−1^ (*v*_4_) while for the AsO stretching region this is found at 849, 835, 822, and 738 cm^−1^.These regions can also be observed in Figure 8, as well as the peak displayed in the region 400–500 cm^−1^, which in the figure is located at 456 cm^−1^. For the case of the band that was reported which corresponds to K-jarosite, this was reported at 1009.8 cm^−1^. Finally, the peaks displayed at 1306, 1548, and 1612 cm^−1^ could be due to the presence of AsO_4_ substituting the SO_4_, due to the similarity to that found for the kankite [(Fe^3+^)_2_(AsO_4_)^.^3H_2_O] at 1469 cm^−1^ and for the tilasite [CaMg(AsO_4_)F] at 1318 and 1518 cm^−1^ [45].

**Figure 9:**
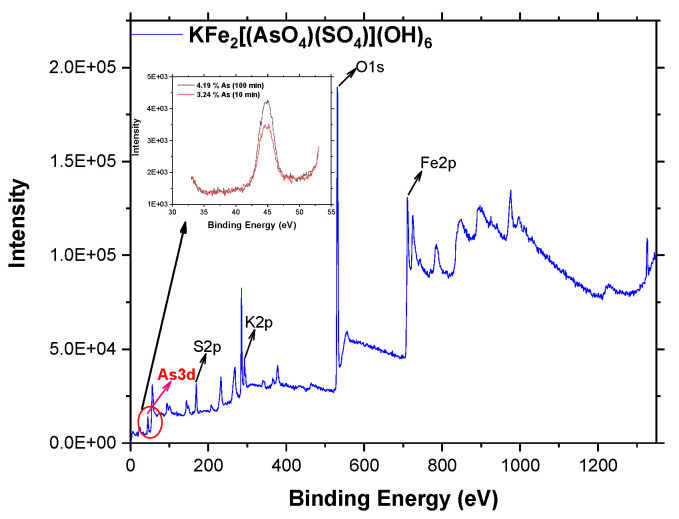
XPS spectra and contents of As(V) at 10 and 100 min after incorporation into jarosite structure.

Finally, to confirm the incorporation of As(V) (at 10 and 100 min) into the structure of decomposed potassium jarosite, the XPS analysis was carried out and the results are shown in Figure 9.

The spectra reveal the presence of Fe, K, S, and O, which are the principal components of jarosite, along with the presence of As3d (at 42–45 eV), confirming the incorporation of this element into the jarosite, as was also reported in other work [46]. In the upper left graph, there are peaks corresponding to As(V) for 10 and 100 min of the process of adsorption, where it can also be observed that for 10 min of reaction, only 3.24% of As(V) was adsorbed, increasing its content up to 4.18% at 100 min of reaction.

## 4. Discussion

Synthesis of potassium jarosite using a modified method resulted in spherical and semispherical particles, as were obtained by other authors [35,41,47]. However, in this case, the particle sizes obtained were smaller (1 to 2 μm) than those obtained in previous works [39,40], which make these powders potentially attractive for multiple usages, due to their major superficial contact area. For this study, these kinds of particles were adequate for the decomposition process and the immediate adsorption of arsenic (V) into the decomposed jarosite, which was carried out at ambient temperature, also promoting its recrystallization at the same temperature. In our case, 4.19% As(V) was adsorbed in the jarosite at 100 min of reaction, while other authors reached only 1.72–3.19% As(V) adsorbed, but at 94 °C, 24 h of reaction, 180–200 °C, and 1 bar of pressure [25,26,27,34,35]. Theoretically, for natural jarosites, the approximate content of As(V) in its structure is about 1.6% [37,38], so the results obtained here are adequate taking into account that the process was carried out at ambient temperature.

In relation to the amount of As(V) adsorbed, it was determined to be a value of 4.19% at 100 min of reaction, and although SEM-EDS (punctual and semiquantitative analysis) obtained an average value of 8.48%, the XPS results are considered more accurate. As a consequence, the obtained results are of importance due to the As(V) adsorption being executed at ambient temperature and with only 2 h of reaction, incorporating the metal into a compact and crystalline structure such as AsO_4_, and substituting the SO_4_, in a stable form, as was reported by other authors [35], where it was demonstrated that As(V) remains in the decomposed structure of ammonium jarosite with As(V). Further, in works related to the bioleaching process of arsenic-rich minerals, it was found that this process could promote the substitution of AsO_4_ for SO_4_, and when the pyrite bioleaching occurs in presence of these minerals, this can result in the oxidation of As(III) to As(V), which also acted as the promoting seed for scorodite crystallization [48], as was found in this work, where AsO_4_ also could promote the recrystallization of decomposed jarosite after As(V) adsorption.

Finally, this work is a preliminary study to determine the ability of synthetic jarosite-type compounds decomposed in alkaline media to adsorb As(V) into their structure and immediately after the adsorption process, to recrystallize, immobilizing these metals. In this sense, the stability of As(V) in the recrystallized structure of jarosite could be due to the formation of ferric arsenates when As(V) is trapped by the Fe contained in the amorphous and porous FeOH structure (obtained after jarosite decomposition). The above was described by other authors; in the first case, with a pre-treatment of an As-bearing eluate from a resin, they achieved stabilization of As(V) with iron salts, forming iron (III) arsenate, and in the second case, the oxidation, adsorption, and stabilization of As was carried out using Fe hydroxides, and this last technology is capable of reaching up to 99.9% in removal efficiency [49,50,51]. In this work, unlike what was mentioned above, the use of resins and Fe-hydroxide reagents can be omitted while also achieving an adequate adsorption and stabilization of As(V) directly into the decomposed jarosite structure.

Following the results of this study, future works will be related to the use of this process using both synthetic jarosites and that precipitated in the zinc industry [46] (considered hazardous waste) to immobilize heavy metals from contaminated water. In the same way, another possible study will relate to the incorporation of these compounds into porous concrete to fabricate filters or blocks to be used in the treatment of water for decontamination of heavy and toxic metals.

## 5. Conclusions

The principal objective of this work was to develop a novel process for the adsorption of As(V) for the treatment of wastewater. According to the results, it is possible to determine that the proposed process is viable and can be used for adsorption of As(V) into the decomposed structure of a synthetic potassium jarosite. The main conclusions obtained in this research are the following:

1. The modified synthesis process, executed at 75 °C and only 9 h of reaction, produces a browning powder of potassium jarosite with spherical and semispherical particles of 1–2 μm sizes, which is of importance for the subsequent decomposition, As(V) adsorption, and recrystallization processes.
2. As(V) adsorption in the decomposed structure of potassium jarosite is possible, attaining 4.19% of As(V) in the jarosite structure. The process was executed at ambient temperature, which is of great interest.
3. During the As(V) adsorption process, it is also possible to recover the original structure of potassium jarosite by decreasing the pH to 1.1 (original pH used for synthesis).
4. This process of As(V) adsorption and recrystallization of jarosite structure could be used with jarosite precipitated in the zinc industry (waste) for the treatment of As(V)-contaminated water.

## Figures and Tables

**Table 1:** Data used for the calculation of average crystallite size of J-K-H synthesized.

Peaks	Intensity %	K	Λ	FWHM	2θ	cos θ (rad)	Crystallite Size (nm)
1	100	1	1.789	0.512	34.108	0.956	3.655
2	53.8	1	1.789	0.471	33.720	0.957	3.969
3	43.1	1	1.789	0.333	20.566	0.984	5.460
4	25.0	1	1.789	0.469	54.094	0.891	4.283
5	22.7	1	1.789	0.464	46.770	0.918	4.201
6	21.2	1	1.789	0.331	28.605	0.969	5.578
7	20.0	1	1.789	0.467	58.882	0.871	4.399
8	11.3	1	1.789	0.369	17.632	0.988	4.906
				Average Crystallite size			4.556

**Table 2:** Average punctual chemical analysis of potassium jarosite synthesized at 75 °C for 9 h (SEM-EDS).

Sample	O (%)	Fe (%)	S (%)	K (%)	H_3_O + OH *
Potassium Jarosite	43.6	20.3	10.2	5.2	21.7

* By difference.

**Table 3:** SEM-EDS analysis performed on synthesized potassium jarosite and that with As(V) adsorption at different reaction times (30, 90, and 120 min).

Sample	% O	% S	% K	% Fe	% As
J-K *	43.60	10.20	5.20	20.30	0
J-K-As-30 min	38.05	9.44	5.31	39.89	7.30
J-K-As-90 min	36.34	9.23	5.14	40.78	8.48
K-K-120 min	37.82	9.70	5.22	38.77	8.47

* Difference corresponds to H_3_O + OH.

## Data Availability

Not applicable.
